# How Do Music Activities Affect Health and Well-Being? A Scoping Review of Studies Examining Psychosocial Mechanisms

**DOI:** 10.3389/fpsyg.2021.713818

**Published:** 2021-09-08

**Authors:** Genevieve A. Dingle, Leah S. Sharman, Zoe Bauer, Emma Beckman, Mary Broughton, Emma Bunzli, Robert Davidson, Grace Draper, Sheranne Fairley, Callyn Farrell, Libby Maree Flynn, Sjaan Gomersall, Mengxun Hong, Joel Larwood, Chiying Lee, Jennifer Lee, Lewis Nitschinsk, Natalie Peluso, Sarah Elizabeth Reedman, Dianna Vidas, Zoe C. Walter, Olivia Renee Louise Wright

**Affiliations:** ^1^UQ Music, Dance and Health Research Group, The University of Queensland, Brisbane, QLD, Australia; ^2^School of Psychology, The University of Queensland, St Lucia, QLD, Australia; ^3^School of Human Movement and Nutrition Sciences, University of Queensland, Brisbane, QLD, Australia; ^4^School of Music, The University of Queensland, Brisbane, QLD, Australia; ^5^University of Queensland Business School, Brisbane, QLD, Australia; ^6^School of Health and Rehabilitation Sciences, University of Queensland, St Lucia, QLD, Australia; ^7^Child Health Research Centre, University of Queensland, Brisbane, QLD, Australia

**Keywords:** music listening, singing, instrumental music, rapping, dance and movement, health, well-being

## Abstract

**Background:** This scoping review analyzed research about how music activities may affect participants' health and well-being. Primary outcomes were measures of health (including symptoms and health behaviors) and well-being. Secondary measures included a range of psychosocial processes such as arousal, mood, social connection, physical activation or relaxation, cognitive functions, and identity. Diverse music activities were considered: receptive and intentional music listening; sharing music; instrument playing; group singing; lyrics and rapping; movement and dance; and songwriting, composition, and improvisation.

**Methods:** Nine databases were searched with terms related to the eight music activities and the psychosocial variables of interest. Sixty-three papers met selection criteria, representing 6,975 participants of all ages, nationalities, and contexts.

**Results:** Receptive and intentional music listening were found to reduce pain through changes in physiological arousal in some studies but not others. Shared music listening (e.g., concerts or radio programs) enhanced social connections and mood in older adults and in hospital patients. Music listening and carer singing decreased agitation and improved posture, movement, and well-being of people with dementia. Group singing supported cognitive health and well-being of older adults and those with mental health problems, lung disease, stroke, and dementia through its effects on cognitive functions, mood, and social connections. Playing a musical instrument was associated with improved cognitive health and well-being in school students, older adults, and people with mild brain injuries *via* effects on motor, cognitive and social processes. Dance and movement with music programs were associated with improved health and well-being in people with dementia, women with postnatal depression, and sedentary women with obesity through various cognitive, physical, and social processes. Rapping, songwriting, and composition helped the well-being of marginalized people through effects on social and cultural inclusion and connection, self-esteem and empowerment.

**Discussion:** Music activities offer a rich and underutilized resource for health and well-being to participants of diverse ages, backgrounds, and settings. The review provides preliminary evidence that particular music activities may be recommended for specific psychosocial purposes and for specific health conditions.

“*Music tells us things – social things, psychological things, physical things about how we feel and perceive our bodies – in a way that other art forms can't”* – David Byrne (2012), How Music Works, p. 101.

The body of research on music, health and well-being has developed rapidly in the past decade, yielding dozens of empirical studies, reviews (Daykin et al., 2018; Sheppard and Broughton, 2020), books (MacDonald et al., 2012; Bonde and Theorell, 2018), and journals such as the *Journal of Music, Health and Well-being, The Arts in Psychotherapy*, and *Arts and Health*. This work has been summarized in ground-breaking reports such as the UK All Party Parliamentary report on creative health (Gordon-Nesbitt and Howarth, 2020) and the scoping review of the role of the arts in improving health and well-being published by the World Health Organization (Fancourt and Finn, 2019). Despite rapid advances in the field, however, there remain some limitations in the literature which this review seeks to address. First, the term “music” has been used to refer to a range of activities, which are at times poorly defined (Kreutz, 2015). Consider the following examples: personalized music listening for pain management in people with fibromyalgia (Linnemann et al., 2015); group singing for adults with chronic mental health conditions (Williams et al., 2019); a hip-hop project for sexual health promotion in Indigenous school students (McEwan et al., 2013); and dance for Parkinson's (Shanahan et al., 2015). All four are examples of music and health projects yet these activities clearly engage distinct physical, social, and psychological processes to achieve improvements in participants' health and well-being. We need to better articulate what type of music activity we are referring to in studies of “music,” and to examine the evidence in relation to the health and well-being effects of specific music activities.

Secondly, research in the music, health and well-being field is often prone to risks of bias arising from methodological issues such as convenience sampling, small sample sizes, lack of control or comparison conditions, and lack of independent assessment (Dingle et al., 2019; Clift, 2020). For these reasons, we will adopt a simple measure of research quality based on guidelines from the British Psychological Society QMiP Guidance for qualitative psychologists (Qualitative Methods in Psychology REF Working Group, 2018) and the Cochrane Risk of Bias 2.0 guidelines (Sterne et al., 2019) for quantitative methods (see Methods section) to ensure that research with a level of quality informs the conclusions of this review.

Third, it is largely unclear *how* such music activities affect health and well-being. That is, what are the processes through which these effects are achieved? It may be the case that different music activities exert their effects through distinct processes. For example, dance for Parkinson's may improve participants' well-being through its effects on gait and synchronized movement whereas music listening for pain management might exert its effect through dampening physiological arousal or providing a distraction. The answers to this important question will help health professionals to make recommendations to individuals and their loved ones about whether a music activity or intervention is likely to help them to manage their health symptoms. Research into the biological mechanisms linking music activities with health and well-being outcomes has been summarized recently. Finn and Fancourt (2018) reviewed 44 studies that involved adults listening to music in clinical and non-clinical settings reported that 13 of 33 biomarkers tested (such as cortisol, blood glucose and immune system measures) were reported to change in response to listening to music, indicating a stress-reducing effect (Finn and Fancourt, 2018). Group singing in low stress conditions such as rehearsals is associated with decreased cortisol while singing in high-stress conditions such as performances has been related to increased cortisol levels (Beck et al., 2000; Schladt et al., 2017). Similarly, group drumming has been associated with a modulation of immune response (Fancourt et al., 2016). Given this existing evidence regarding the biological mechanisms, in this scoping review we will focus instead on the psychological (e.g., emotional, cognitive, behavioral, motor) and social (e.g., bonding, inclusion, identity, cultural) processes that might explain the health and well-being effects of music activities.

In planning the scoping review, we searched for a comprehensive theoretical model that would account for a spectrum of musical activities and health and well-being outcomes, through a range of psychosocial processes. Unfortunately, the field of music, health and well-being lacks a widely established and comprehensive framework (Dingle et al., 2019). Our conceptualization is aligned with the contextual model by Kreutz (2015) showing the beneficial effects of musical activities on well-being and quality of life. According to this model, engagement in a musical activity provides individuals with a new context in which to interact and the combination of individual and contextual variables evoke self-regulatory processes at conscious and/or subconscious levels. Examples of such processes shown in the model include modifying cognitions, emotions, and actions by strengthened self-regulation. The consequences are often an improvement of psychological well-being and other positive outcomes (Kreutz, 2015). The boundaries of how this works (to what degree, for how long, and for whom) remain unclear and subject to ongoing hypothesis-driven research.

We also drew variables from three other models in developing search terms for the review. The first was the Therapeutic Music Capacities Model (Brancatisano et al., 2020) which links individual properties of music to “therapeutic mechanisms,” leading to cognitive, psychosocial, behavioral, and motor benefits. Some of the therapeutic mechanisms specified in the TMCM are conceptually relevant to populations with neurological disorders for whom the model was developed but are rarely assessed as part of music intervention research (e.g., neuroplasticity, mirror neuron systems, auditory motor coupling, and neural entrainment). We adopted other mechanisms that are more commonly assessed in music research as some of our search terms, such as arousal, mood, and memory. Another model that informed the review is the BRECVEMA model (Juslin et al., 2010) which describes eight mechanisms by which music listening influences emotional responses, in addition to cognitive appraisal. BRECVEMA is an acronym for Brain stem reflex, Rhythmic entrainment, Evaluative conditioning, Contagion, Visual imagery, Episodic memory, Musical expectancy, and Aesthetic judgement. This model is most suitable for experimental music listening research, however, it can be argued that some of these mechanisms apply to other music activities (e.g., rhythmic entrainment may occur as part of instrumental music playing, dance, and rapping). A third model that has been applied to health interventions in groups is the social identity approach (Tajfel and Turner, 1986; Turner et al., 1987; Jetten et al., 2014). According to this model, to the extent that participants identify with their group, they may access psychological resources from the group such as support, meaning, control and self-esteem. This model has been shown to explain the health and well-being effects of group singing (Williams et al., 2019; Dingle et al., 2020; Tarrant et al., 2021) and other music activities such as dance and instrumental music groups (Kyprianides and Easterbrook, 2020; Draper and Dingle, 2021). From this model, we drew social connection, self-esteem, and identity as processes.

## Methods

The scoping review was conducted by an interdisciplinary group of academics and students from the Schools of Psychology, Music, Human Movements and Nutrition, Physiotherapy, Business, and hospital based Clinical Research Centers at the University of Queensland during February to December 2020. We formed into small working parties of two to four people, each focusing on one of the eight music activity categories. Reliability was established by two or more members of each working group screening the same 50 abstracts in their category and meeting to ensure that the selection criteria were applied consistently. Following this, the remainder of abstracts were divided up among group members for screening. This process resulted in too many papers for inclusion in the full review, so the authors agreed to re-screen the “included” abstracts by applying our quality criteria to exclude all but the best quality research in each category. Importantly, studies in which the intervention was clearly music therapy or a form of psychotherapy were not included, as these bodies of research have been reviewed elsewhere. The literature search was conducted using nine search engines: CINAHL, Embase, Music Periodicals, PsycInfo, PsycNET, PubMed, Scopus, SPORTDiscus, and Web of Science. Search terms are available from the corresponding author on request. Inclusion criteria were that the papers report on empirical research (not reviews or theoretical papers), published in the English language, involving adult participants, and reporting on a health or well-being measure as well as one or more psychological or social process measures that we refer to as “mechanisms” (note that the authors of the studies did not necessarily regard their study design in this way). For quantitative studies, the following criteria were applied:

a. The study used psychometrically validated measures of a health or well-being outcome and at least one process variable.
b. The study had at least 20 participants per condition^1^.
c. If a control or comparison condition was included, allocation of participants to conditions was randomized or a check was done to ensure that the two subgroups were comparable at the start of the study.
d. Assessors were independent of the people delivering the music activity (to avoid demand characteristics on participants' responses).

For studies using qualitative methods, the following quality checks were applied:

e. A description and explanation for the type of analysis was given.
f. There was independence between the facilitators of the music program and those collecting and analyzing the data (or involvement of an independent coder in the analysis).

## Results

### Overview of the Studies

The number of papers at each stage of the scoping review are shown in Table 1. Detailed descriptions of the participants, design and intervention, process measures, health or well-being outcomes, and a summary of the results of each study are presented in Supplementary Table 1. As would be expected, the health and well-being outcomes varied across the musical activity categories. In the receptive music listening studies, pain and indicators of post-operative recovery were common outcomes. In the studies of intentional music listening, pain was again a common outcome, as well as health behaviors such as exercise, symptom checklists and measures of well-being, health related quality of life, and patient satisfaction. In the music sharing studies, outcomes included pain, fatigue, agitated and aggressive behavior, quality of life, and well-being. The instrument playing studies reported health outcomes including cognitive health in older adults, health behaviors, social determinants of health (housing stability and criminal behavior), and well-being. In studies of group singing, the outcomes included mental and physical health, cognitive health, well-being, and quality of life. Studies of movement and dance reported outcomes for cognitive health, healthy weight, mental health, and quality of life. Studies of lyrics and rapping reported outcomes such as mental health and cognitive health. Finally, the studies of music composition, songwriting and improvisation included outcomes such as well-being and cultural determinants of health.

**Table 1:** Flow of decisions about papers through the abstract screening, quality screening, and full text review process.

	**Receptive Music listening**	**Intentional Music listening**	**Sharing music (live, recorded)**	**Solo or group instrument playing**	**Group singing**	**Music and movement; dance**	**Lyrics; rapping**	**Song-writing; Composition; improvisation**	**Total**
Total entries	2,777	1,546	2,128	1,921	1,886	870	2,785	1,567	15,480
No. after duplicates removed	1,922	1,226	1,478	1,701	1,455	743	1,978	1,280	11,783
No. for full text review	34	35	13	12	146	82	17	11	350
Final no. included	11	12	5	10	14	4	4	3	63

Process measures included arousal, emotion or mood, cognitive measures (e.g., memory, attention), self-esteem/achievement, physical activation, social connection, and identity. Most of the studies in the receptive music listening category and some of the intentional music listening studies reported on psychophysiological measures of arousal, such as blood pressure, heart rate, respiratory rate, and skin conductance. Although these could be viewed as biological measures (which was not the focus of the review or search terms), they are also commonly used in experimental psychological research as indicators of emotional arousal. For this reason, we kept these studies in the review. A summary of the process variables supported by the literature in each musical activity category is presented in Table 2 and explored in further detail in the following sections.

**Table 2:** Summary of available evidence about the psychosocial mechanisms by which music activities affect health and well-being.

**Mechanism**	**Receptive music listening**	**Intentional music listening**	**Shared music listening (live, radio)**	**Instrumental music playing**	**Group singing**	**Lyrics; rapping**	**Music and movement/dance**	**Composition/songwriting/improvisation**
Physiological arousal	+/–	+/–		+				–
Emotion/mood	+	+	+	+	+	+	+	+
Cognitive (e.g., memory)		+	+	+	+	+	+/–	
Self-esteem/achievement				+	+	+		+
Physical activation	+	+	+	+		+	+/–	
Social connection	+		+	+	+	+	+	+
Identity			+		+	+	+	+

*Key: +, studies reviewed showed positive evidence; +/–, some studies reviewed showed positive evidence some studies found no evidence; –, studies reviewed found no evidence; blank means the studies reviewed did not measure this*.

### Receptive Music Listening

There is some conceptual overlap between receptive music listening and intentional music listening (next section). We divided studies into the two categories based on the idea that receptive music listening involved participants being in places where music is playing but they were not involved in the music selection process whereas intentional music listening involved some degree of participant engagement in the choice of music they listened to. Among the receptive listening studies, there were 1,922 abstracts screened, 78 selected for full-text review, of which 11 met the criteria for inclusion (see Table 1). Nine studies were conducted in a medical setting and investigated the effects of music listening before, during, or after a medical procedure. These included dental procedures, elective surgery, and breast biopsy. Two studies examined the impact of background music on patients with severe dementia (Götell et al., 2002; Gotell et al., 2009). Methods of receptive listening generally utilized assorted ‘background music’ that was played for participants. These were described as instrumental or classical (Calcaterra et al., 2014; Franzoi et al., 2016; Kipnis et al., 2016; Seinfeld et al., 2016; Çetinkaya et al., 2018), new age (Kipnis et al., 2016; Téllez et al., 2016), relaxing (Twiss et al., 2006; Nilsson, 2009), or participant selected music (Twiss et al., 2006).

Across the studies in medical settings, health outcomes primarily focused on pain, recovery from operations, and patient satisfaction. The mechanism of these effects appeared to be the reduced levels of anxiety, distress, and increased relaxation among patients listening to background music compared to the control groups, who were generally in silence. Reductions to pain were less clear among children in one study where there were some age-based differences in pain reports, where older children showed more pain amelioration (Calcaterra et al., 2014). This may also have been due to older children being better able to understand the pain scale. In the two studies of older adults with dementia, both publications reported from the same study observing nine patients and carers undertaking a morning routine over three conditions: usual morning care, morning care with familiar background music, and caregiver singing. Results found that background music was related to patient functioning, well-being and decreased aggressive behaviors through its effects on physical activation, increased bodily and on sensory awareness, and a strengthened ability to carry out daily living tasks. Patients showed more agency and playfulness in their interactions with their carers, demonstrating improved social connection and interactions.

### Intentional Music Listening

Following screening of 1,226 abstracts, 12 studies met the inclusion criteria for the review of intentional music listening research (described in Table 1). Methods of intentional listening across all studies utilized either researcher provided music and/or participant preferred music during the music listening interventions. Intervention lengths varied for each study and ranged from a single session of music listening (e.g., Särkämö et al., 2008) to 6 months (e.g., Clark et al., 2016). The way in which music listening was applied as an intervention was also mixed with some research emphasizing music listening during periods where participants were undergoing treatment or experiencing symptoms (O'Callaghan et al., 2012; Mercadíe et al., 2015), during recovery from health procedures (Särkämö et al., 2008; Drzymalski et al., 2017), or during specific daily activities, such as walking or relaxing (Clark et al., 2016; Helsing et al., 2016).

Health outcomes included pain, fatigue, health behaviors such as exercise, symptom checklists and measures of well-being, health related quality of life, and patient satisfaction. Music listening appeared to produce such outcomes through its effect on emotions regulation where several studies reported a reduction in feelings of distress, including specific measures of depression, anxiety, stress (Särkämö et al., 2008; Helsing et al., 2016; Sorensen et al., 2019); greater feelings of relaxation and nostalgia (Clark et al., 2016; Helsing et al., 2016; Kulibert et al., 2019; Sorensen et al., 2019); improved mood and reduced agitation (Clark et al., 2016; Ihara et al., 2019) (see Supplementary Table 1). The duration of these effects is difficult to ascertain due to the varying lengths of follow up across measures and studies. However, one study found that reduced levels of anxiety and pain were sustained for at least 12 h after music listening (Fernando et al., 2019). Several of these studies reported positive emotional effects of music listening compared to a control (no music listening group). However, two studies compared music listening with other active sound or meditation conditions and neither found differences between active conditions (Mercadíe et al., 2015; Sorensen et al., 2019). These studies lacked a no-music control condition, making it difficult to form robust conclusions about the efficacy of intentional music listening in these studies. Similarly, a study of 169 young people with at least mild psychological distress using a music and emotion regulation mobile phone app showed no differences on emotion regulation, distress, or well-being between the music listening and the waitlist group at 1 month follow up (Hides et al., 2019).

Cognitive mechanisms measured in the intentional music listening studies included measures of attention and verbal memory among stroke patients, which were better amongst music listeners compared to those who listened to audiobooks or controls (Särkämö et al., 2008) (see Supplementary Table 1). Another study took behavioral observations of music recognition and ability to follow rhythm among people with dementia (Ihara et al., 2019). Evidence for physical activation was limited to two studies. One used behavioral observations of people with dementia (Ihara et al., 2019) and revealed that intentional music listening increased expressions of joy, eye contact, eye movement, engagement, talkativeness, and moving/dancing. Similarly, a study of participants with cardiac disease (Clark et al., 2016) reported that listening to music while walking made them feel more energized and the music tempo influenced them to walk faster or maintain an enhanced pace, motivated them to move and some found it helped them to walk for longer periods.

### Sharing Music

1,478 abstracts were reviewed with only five studies about music sharing meeting the inclusion criteria for full review. Only one study did not use live music, instead utilizing scheduled Radio programs to initiate music sharing across people's homes (Travers and Bartlett, 2011). These studies tended to report outcomes on well-being, quality of life, pain and agitated behavior. The processes by which shared music listening appears to achieve these outcomes was through emotion, cognition (memory), physical activation (synchrony), social connection, and a sense of identity (see Table 2). The strongest results were for improved mood and/or emotions, which were found to improve for shared music listening across all studies. Improved social interaction and communication also appeared to show consistent effects, which were particularly marked among participants with dementia, though less so for those with more severe dementia (van der Vleuten et al., 2012; Clements-Cortés, 2017; Shibazaki and Marshall, 2017; Toccafondi et al., 2018). As part of this, sharing music stimulated participants' memories and facilitated reminiscing and storytelling that were shared with musicians, staff, and family members. In contrast, music sharing through community radio programming found no changes to loneliness among this shared listening group, likely indicating that less social interaction was facilitated (Travers and Bartlett, 2011). These results imply that there is something unique about sharing music when in the physical presence of others.

Synchronized movement and physical activation increased during live music sharing alongside the ability to remember, cognitively perceive, and anticipate auditory musical elements (Clements-Cortés, 2017; Shibazaki and Marshall, 2017). Participants were reported to be clapping, singing, and generally moving to the music. Shibazaki and Marshall (2017) noted that these physical responses were even evident for people with mobility issues and among those who had suffered strokes. Finally, for people with dementia, even when dementia was advanced, carers and researchers observed clear cognitive effects while sharing music, such as participants being able to predict, anticipate, and expect different musical patterns and changes (Shibazaki and Marshall, 2017).

### Instrumental Music

From 1,701 abstracts screened, nine studies of instrumental learning and playing met selection criteria for full review. These focused on health and well-being outcomes from musical instrument playing, such as cognitive health in older adults, health behaviors, social determinants of health (housing stability and criminal behavior), and well-being. Instrument playing was associated with these outcomes *via* its effects on cognitive, mood, and/or social processes (see Supplementary Table 1 and Table 2). Collectively, the research found that playing an instrument resulted in several positive outcomes, including improved mental health and quality of life and well-being (Perkins and Williamon, 2014; Seinfeld et al., 2016). Music instrument learning also resulted in improved enthusiasm, happiness, relaxation, and tolerance of uncertainty among people with learning disabilities (Wilson and MacDonald, 2019). Being part of a band or music group improved perceptions of social support and actual participation in social activities, interpersonal communication, self-esteem, and self-confidence among long-term musicians (Knapp and Silva, 2019), new musicians (Perkins and Williamon, 2014) and people with learning difficulties (Wilson and MacDonald, 2019). This latter study found that people who were socially isolated were more difficult to engage in music groups, with participants reporting lower levels of confidence and self-esteem (Wilson and MacDonald, 2019). Self-efficacy scores among children learning a musical instrument were also higher among those learning compared to those not learning an instrument, with this effect higher for girls, compared to boys (Ritchie and Williamon, 2011). This self-efficacy was related to greater levels of well-being and higher pro-sociality, with self-efficacy heightened particularly for girls.

Physical activation was found to be related to self-efficacy among children, where self-efficacy for music learning was associated with less hyperactivity, emotional symptoms, and behavioral problems (Ritchie and Williamon, 2011). Among older adults with higher SES, those learning to play a musical instrument reported a greater increase in the frequency of behaviors promoting physical activity and spiritual growth than older adults in the comparison condition (a U3A shared learning project) (Perkins and Williamon, 2014). Cognitive mechanisms were measured across several studies and found that for older adults, playing instruments was related to improvements in cognitive processing speed and attention, verbal fluency, executive function, visual scanning, and motor ability (Bugos et al., 2007; Bugos and Kochar, 2017), as well as letter fluency, learning, and short-term memory (Mansens et al., 2018). One study used fMRI in people with mild traumatic brain injuries following 8 weeks of piano lessons and found that there was a change to activation of the medial orbitofrontal cortex (OFC) (Vik et al., 2018). The OFC network regulates higher order cognitive processing, such as executive functions, including attention, decision-making, impulse control, and social behavior.

### Group Singing

A total of 1,455 abstracts were identified in the initial search from which 14 studies met selection criteria for the full review, including six qualitative and eight quantitative studies (see Table 1). Prominent outcomes included mental health and well-being, cognitive health, and lung health. Group singing appeared to produce these health and well-being effects through the social, emotional and physical processes. Choral rehearsals have been found to increase feelings of social inclusion and connection over the duration of a singing rehearsal (see Supplementary Table 1). Studies involving both small and large group choirs of up to 232 members found that singing fosters social closeness, even in large contexts where individuals are not known to each other (Weinstein et al., 2016). Even after a single session of singing, a large group of unfamiliar individuals can become bonded to the same level as those who are familiar to each other within that group. These social inclusion effects are particularly important for various marginalized groups. For instance, 50 minority African Canadian women living in Nova Scotia identified choir singing and listening to spiritual music as spiritual activities that helped protect against the psychological effects of racism (Beagan and Etowa, 2011). The women described how singing supported their physical and mental health through a spiritual connection with the Lord and through their cultural connection with the African Christian community. In another study, women from nine different nationalities living in the UK who experienced postnatal depression participated in a 10-week singing group and reported that the sessions provided an authentic, social and multicultural creative experience (Perkins et al., 2018). Two Australian studies involving adults who were marginalized due to chronic mental and physical health problems described how choir singing helped them to develop social connections within the choir (Williams et al., 2019) and later with audiences (Dingle et al., 2013). Furthermore, a reduction in loneliness and an increased interest in life was reported by an ethnically and racially diverse group of seniors participating in a Community of Voices choir in San Francisco (Johnson et al., 2020).

Cognitive, social, and mood effects of group singing are prominent in older adult studies (Lamont et al., 2018). For example, in retirement village residents in Australia, those who attended an 8-session group music program called Live Wires showed significantly improved cognitive performance and identification with the retirement village compared with the control group (Dingle et al., 2020). Similarly, in the Singing for the Brain project in the UK, interviews with 20 people with dementia and their care givers indicated that important mechanisms were cognitive (accepting the diagnosis, positive impact on memory), social (a shared experience, feeling included and supported), and improved mood (Osman et al., 2016). Similarly a study in Finland assessed people with dementia and their caregivers before and after a 10-week program of either singing or music listening together, designed to coach the caregivers to incorporate music and singing into their dementia care (Särkämö et al., 2014). Music listening temporarily improved overall cognition, attention and executive function, and a longer-term improvement in orientation, while singing enhanced short-term and working memory. Music listening had a long-term positive effect on Quality of Life for both the patients and caregivers.

In terms of physical mechanisms, participants of the Sing for Lung Health choir described improvements in breathing, sputum clearance and exercise tolerance, as well as a general sense of improved well-being. Again, social connections and a shared purpose were key mechanisms, as well as physical activation (McNaughton et al., 2017). This 12-week program featured deep breathing, vocalization exercises and singing rounds of familiar songs.

A sense of achievement and a new identity as a member of a choir were mechanisms revealed in several studies (Dingle et al., 2013; Perkins et al., 2018; Williams et al., 2020), particularly during performances (McNaughton et al., 2017). Singing, however, is not necessarily better than other arts-based group activities in terms of health and well-being effects. A study 135 adults involved in seven different adult education classes in singing, creative writing and crafts found that mental and physical health, and satisfaction with life, improved in all groups (Pearce et al., 2016). In the study with marginalized adults, mental well-being improved for members of both a choir and a creative writing group as long as participants formed a sense of identity with their group (Williams et al., 2019).

### Music, Movement, and Dance

This search retrieved 743 articles of which four studies met criteria for full review. The health outcomes measured differed widely across the four studies. These included improved measures of cognitive health in the participants with mild cognitive impairment (Doi et al., 2017); healthy weight measures (BMI and % body fat) of African American women (Murrock and Gary, 2010); improved cognitive health among stroke survivors (Jeong and Kim, 2007); and mental health of new mothers (Vlismas et al., 2013).

While social connection was acknowledged as an important process across most of these studies, only two measured types of social connection. Interventions were found to improve the quality of interpersonal relationships for stroke survivors compared to people who did not participate in movement interventions (Jeong and Kim, 2007), and to improve interactions between mothers and their infants (Vlismas et al., 2013). Specifically, mothers felt that they enjoyed interactions with their infants more and reported increases in dyadic reciprocity between them. Similarly, physical activation, while acknowledged as a driving mechanism, was only measured in two studies. For African American women, participating in a dance group meant that they were more physically active than those not participating in dance (Murrock and Gary, 2010). However, for adults with mild cognitive impairment, there was no difference in physical activity levels whether they were part of the dance group, playing instruments, or in a health education group. For one study, cultural identity was made salient for the participants, where African American women reported that the dance intervention and choreography incorporated the importance of their church, spirituality, values, and beliefs and provided a positive space for them to talk about their health concerns (Murrock and Gary, 2010).

### Lyrics and Rapping

From 1978 abstracts reviewed, four articles focusing on rapping or other lyric-focused music activities met our inclusion criteria. The outcomes from these included mental health, well-being, and cognitive health. The effects of lyrics and rapping appeared to act on emotional and social processes, self-esteem and identity (see Supplementary Table 1 and Table 2). For instance, for children and adolescents, sung or spoken lyrics (including rap), resulted in improvements to measures of emotional well-being on the Strength and Difficulties Questionnaire (Uhlig et al., 2019) and teacher-rated emotional symptoms, empowerment, and fewer depressive symptoms (Travis and Bowman, 2012). Further, those least likely to report depressive symptoms were those who felt rap music inspired them to better connect with others, consider the experiences of others, and want to make a difference in their communities. Young people listening to rap and hip-hop showed that their sense of cultural identity was associated with music-based empowerment (Travis and Bowman, 2012), and physically engaging in rap and song among children influenced their levels of physical activation (Uhlig et al., 2019). This included reductions in hyperactivity and inattention, and improved goal-directed behavior (Uhlig et al., 2016). Sleep time also showed changes among this group those in the rap and sing group slept significantly more than children who did not participate in this program.

A study in university students found that exposure to lyrics related to suicide were associated with remembering more nihilistic lyrics than were present in the song (Peterson et al., 2008). However, after exposure to this music, many participants responded with stories that exhibited altruistic themes. Higher endorsement of lyrical messages around risk (e.g., violence, substance use, and derogatory treatment of women) was related to high self-esteem among young males (Travis and Bowman, 2012). For people with Alzheimer's disease and healthy older adults, memory was positively affected when they were exposed to lyrics that were spoken or sung (Simmons-Stern et al., 2012). For these older adults, both types of exposure to lyrics resulted in equal memory of a songs content.

### Song Writing, Composition, and Improvisation

This search retrieved 1,280 articles, of which only three studies met the inclusion criteria for the review. Music composition was found to be an important tool for supporting healthy aging and well-being of older adults learning to compose music collaboratively with a string quartet and a professional composer. For these participants, composition also created more opportunities for creativity and feelings of control and self-efficacy (Habron et al., 2013) (see Supplementary Table 1). In the study by Bartleet et al. (2016), jamming and music making between Aboriginal and non-Indigenous musicians provided opportunities to develop deep, transformative, intercultural engagement and connection. For these groups, music making was a way to cross boundaries using music as a shared language and to understand and share in diverse experiences. Music students found that the simple act of jamming helped to build a strong rapport, sense of mutual respect, and life-long friendships. Identity making and relationships were very clear among the group-based song writing and composition studies. For example, older adults felt composition led to self and social identity making, and meaningful social engagement with other participants and musicians with some relationships enduring after the program ended (Habron et al., 2013).

In Fallon and colleagues' experimental study (2020), 105 university students were asked to complete a stressful task and were then randomly assigned to one of three recovery conditions: control, music listening, or music improvisation using a xylophone. The physiological measure (electrodermal activity) showed greater stress reduction during recovery for those in the music listening condition compared to the improvisation and control groups (Fallon et al., 2020). The improvisation group showed a significant improvement in self-reported levels of calmness, irritation (decrease), and satisfaction during the recovery phase.

## Discussion

This scoping review of 63 studies revealed that all eight categories of music activities demonstrated some benefits to health or well-being, although it is difficult to make generalized statements due to the diversity of study designs and measures across studies. An abundance of studies of music listening, group singing, and instrument playing met criteria for inclusion, but relatively few focused on music sharing, dance or movement to music, lyrics or rapping, or songwriting, improvisation and composition. As the descriptions in Table 1 indicate, some music activities featured in more than one category (e.g., music listening was involved to some extent in all eight types of activity, apart from some kinds of lyrics/rapping), while other activities were found in only one or two categories (e.g., movements *to* music were a key part of the movement and dance category, while movements *to create* music were characteristic of the instrument playing, group singing, and songwriting, composition, and improvisation category). The eight activities also represent a spectrum of engagement with the selection and creation of music, from very low levels in the case of receptive music listening through to very high levels in the case of songwriting, composition, and improvisation. The purpose of the music activity and the measures assessed in each study reflected this spectrum of engagement. By considering this full spectrum of music activities, the current review extends on previous reviews that had a narrower focus such as music listening (Finn and Fancourt, 2018), group singing (Williams et al., 2018), or instrument playing and dance (Sheppard and Broughton, 2020). It also highlights the need for future research in the field of music, health and well-being to clearly articulate the type of music activity under investigation (Kreutz, 2015).

In regard to the mechanisms by which these music activities produce effects on health or well-being, Table 2 summarizes the evidence drawn from the 63 papers reviewed. Receptive music listening tended to be used in health or medical spaces for the purpose of decreasing perceptions of pain and anxiety and for acute post-operative recovery, or in aged care settings for increased activation and improved mood among older adults with dementia. Many of these studies showed that *decreased physiological arousal* was a key mechanism by which music listening was related to effects on pain and anxiety. The most consistent results were lowered blood pressure, increase in oxytocin, and decrease in cortisol during music listening. Interestingly, music listening was associated with *increased arousal, activation*, and social interaction in the studies of people with dementia and their carers (Götell et al., 2002; Gotell et al., 2009). Of the 13 studies in this category, four measured pain outcomes, and three of these reported lower pain in the music condition (Calcaterra et al., 2014; Téllez et al., 2016; Çetinkaya et al., 2018) while one study did not find any effect of music listening on pain (Chantawong and Charoenkwan, 2017). It is possible that in this study, the researchers' selection of Western or New Age instrumental music did not align with the Thai women's personal preferences during the cervical excision procedure. These findings align with an earlier review showing how music listening can enhance medical treatments and can be used as an adjunct to other pain-management programs (Bernatzky et al., 2011). This review concluded that musical pieces chosen by the patient are typically more effective for pain management than music chosen by a staff member. Interestingly, a recent study found that the music people chose to manage pain was commonly high energy, danceable music with lyrics (Howlin and Rooney, 2020) so it should not be assumed that people select soft, slow tempo, instrumental music when in pain.

Positive effects on *mood or emotion regulation* were reported in studies across all music activity categories (Supplementary Table 1). In the music listening categories, reductions in anxiety were commonly reported. These positive effects on anxiety and pain were not confined to music listening since comparison conditions also produced benefits. For example, a comparison hypnosis condition was associated with decreased anxiety and increased optimism in women undergoing breast tissue biopsy in a hospital clinic (Téllez et al., 2016) while silent relaxation was as effective as music listening for lowering cortisol and pain in knee replacement surgery patients (Finlay et al., 2016). Similarly, the 14 studies on intentional music listening commonly focused on the role of music in reducing distress, particularly in preparation for, during, or recovery from, significant health events. These studies revealed that actively listening to music showed effects on *cognition, emotion, physical activation, and physiological arousal*. These findings are consistent with an established body of research on music listening and affective responses on the two dimensions of arousal and valence (e.g., Juslin et al., 2010; Eerola and Vuoskoski, 2013). The mood enhancing effects of group music activities such as singing, dancing and instrument playing is consistent with the findings of a systematic review of the effects of social group programs (music groups and others) on depression (Dingle et al., 2021) and an earlier longitudinal study of 5,055 UK older adults showing that more group memberships measured in the first wave was associated with a decreased likelihood of depression up to 4 years later (Cruwys et al., 2013).

Enhanced *social bonding and connection* was found in studies across many of the music activity categories. For example, shared music listening in the form of live music concerts enhanced social connections and mood in older adults and in hospital patients, yet was featured in few studies, which suggests this is an underutilized approach within aged care and hospital services. Group singing was associated with health and well-being of older adults and those with mental health problems, lung disease, stroke, and dementia through its effects on cognitive functions, mood, social connections, and identity. Both music listening and carer singing decreased agitation and improved posture, movement, and well-being of people with dementia. These findings indicate that singing is not only beneficial for the identified patients but also for their caregivers and loved ones (Forbes, 2020). *Social and cultural identity* was another mechanism highlighted in relation to some music activities. The finding that identification with a music group is associated with the satisfaction of various psychological needs has been noted in several recent studies (Williams et al., 2019; Kyprianides and Easterbrook, 2020; Draper and Dingle, 2021). Singing, dancing, and hip-hop can help ethnic minority group members to connect with their culture (Murrock and Gary, 2010; Beagan and Etowa, 2011; Travis and Bowman, 2012).

*Cognitive mechanisms* such as improved memory or attention were noted in several music activity categories. For instance, group singing was associated with improved cognitive health in older adults and those with dementia. Learning to play a musical instrument was associated with cognitive performance, self-esteem, and well-being in diverse populations including school students, older adults, and people with mild brain injuries. Dance and movement with music programs were associated with improved health and well-being in people with dementia, women with postnatal depression, and sedentary women with obesity through various cognitive, physical, and social processes. Clinicians and care workers planning to introduce a musical activity to enhance the cognitive health of their participants should consider the level of musical training and capability of new learners. It may be necessary to develop innovative ways for participants to engage with music that do not require an ability to read sheet music or to have a high level of fine motor skill. Group singing can be conducted using lyric sheets and a call-and-response style for learning the various vocal parts, as has been used successfully with marginalized adults (Dingle et al., 2013; Williams et al., 2019). Furthermore, innovative work is in progress adapting musical instruments so that they are simpler for older adults to create music with (MacRitchie and Milne, 2017).

Finally, *self-esteem, empowerment, and sense of achievement* were mechanisms by which rapping, choir singing, musical instrument playing, and composition, songwriting and improvisation produced positive effects on the health and well-being of participants. Rapping, songwriting and composition helped marginalized people to find their voice and increased social inclusion, intercultural connections, and empowerment.

## Conclusion

Although the field of music, health and well-being requires further development, there is emerging evidence that specific music activities may be recommended for specific psychosocial purposes and for specific health conditions. Music activities offer a rich and underutilized resource for health and well-being to participants of diverse ages, backgrounds, and settings.

## Author Contributions

GD designed the scoping review and led the write up. LS generated the search terms, conducted the library searches, assisted with the tabulation of results, and the write up. All authors contributed to the screening, reviewing, and summarizing of papers in their sections and contributed to the final manuscript.

## Conflict of Interest

The authors declare that the research was conducted in the absence of any commercial or financial relationships that could be construed as a potential conflict of interest.

## Publisher's Note

All claims expressed in this article are solely those of the authors and do not necessarily represent those of their affiliated organizations, or those of the publisher, the editors and the reviewers. Any product that may be evaluated in this article, or claim that may be made by its manufacturer, is not guaranteed or endorsed by the publisher.

## Footnotes

^1^This was based on power calculations showing that a sample of 24 is required for a within subjects (pre-post design) ANOVA to find a small effect size with a power of 0.8; while a total sample of 34 (17–18 in each condition) is required for a two-group comparison ANOVA with two assessment points to find a small effect size with a power of 0.8. Our guideline of 20 per condition was chosen to balance the pragmatics of fitting groups of people into halls and spaces where music activities typically take place with the need to design studies with sufficient power to detect an effect if one existed.
