# Supplementary material for: How Do Music Activities Affect Health and Well-Being? A Scoping Review of Studies Examining Psychosocial Mechanisms
Source: Front Psychol. 2021 Sep 8;12:713818. doi: 10.3389/fpsyg.2021.713818 (PMC8455907; doi:10.3389/fpsyg.2021.713818)
Supplement: Supplementary file 1 [file Table_1.docx]

Supplementary Table 1

Description of the 63 studies included in the scoping review, listed by first author surname, within music activity categories.

| Reference | Participant information | Study design and intervention | Process variable(s) | Health or wellbeing outcome(s) | Summary of results |
| --- | --- | --- | --- | --- | --- |
| Receptive Music Listening |  |  |  |  |  |
| 1.Calcaterra et al., 2014 | N: 42 (40 boys and 2 girls), 21 in each condition; mean age 6.7 ± 4.1 years; having day surgical procedures | Design: two groups (Music or Control) x 4 assessment points: admission (T0), end of surgical procedure (T1), awakening (T2), and readmission to the Unit (T3). Quantitative. Country: Italy; Setting: Pediatric surgical unit in hospital. Total duration music: 20 minutes. | Heart rate  Blood pressure  O^2^ saturation  Glucose levels  Cortisol levels | Post-operative distress measured by reactions to pain on Face, Legs, Activity, Cry, Consolability (FLACC) Pain Scale | 20 minutes music listening during awakening from anesthetic dampened the increase of systolic and diastolic blood pressure. Glycemia levels increased in control group and plateaued for the music group. Music had a positive impact on reactions to pain using the FLACC scale. No difference in pain intensity score was noted between the two groups at the end of T2 and T3. A higher number of children reported no or mild pain at T3 in the music group. |
| 2.Cetinkaya et al., 2018 | N: 171 patients undergoing angiography; 91 intervention (58 males), 80 control group (40 males); mean age = 66.8 ± 1.21 years; no analgesic or anxiolytic med. | Design: two group RCT. Quantitative. Country: Turkey; Setting: Hospital cardiovascular surgery unit. Intervention length: 15–20 min on average | Heart rate,  Temp,  Respiration rate,  Systolic BP,  Diastolic BP, State Anxiety (STAI-S) | Pain level estimated using Visual Analogue Scale. | During angiography procedure, patients’ systolic blood pressure, and mean intensity of pain was significantly lower in the music group than the control group. However, there were no differences between groups in anxiety, or any other physiological measures. |
| 3.Chantawong & Charoenkwan, 2017 | N: 150 women undergoing LEEP cervical excision. 74 in music condition, mean age = 46.5 years, 76 in Control condition, mean age = 44.0 years. | Design: RCT with Music or Control; assessments taken at start of procedure and 10 minutes after completion. Country of intervention: Thailand. Setting: Hospital outpatients. Intervention length: from time of arrival until procedure completed. | Anxiety (visual analogue scale) | Pain and satisfaction (visual analogue scales) | For this sample of Thai women, there was no significant effect of listening to relaxing western-style instrumental music on ratings of anxiety, pain, or satisfaction during a cervical excision procedure. |
| 4.Franzoi et al., 2016 | N: 52 (approx. 80% males across conditions); Age: Pre-school and school aged (3-12 years); Sample: pre-operative for a range of surgical procedures | Design: 2 group RCT: Music or Control (usual toys and family members). Country of intervention: Brazil; Setting: Pediatric surgical unit at a public hospital. Intervention length: Single session for 15 mins | Heart rate,  Respiration rate,  Blood pressure,  O^2^ saturation | Yale Preoperative Anxiety scale behavioral domains of activity, vocalization, emotional expression, and interaction with relatives | Pre-operative children showed a significant reduction , specifically in the YPAS behavioral domains of activity, vocalization, emotional expression, and apparent awakening state but not in interaction with relatives after 15 minutes of music listening. This reduction in anxiety was not found in children assigned to the Control condition. |
| 5.Gotell et al., 2002 | N: 9 patients with severe dementia (average age = 84 years) and 5 professional care givers recorded on 27 occasions | Within-subjects design with 3 conditions: usual morning routine, routine+carer singing, and routine+familiar music listening. Qualitative analysis of video recordings. Country: Sweden. Setting: Special care unit for people with severe dementia | Posture,  Motion,  Sensory awareness | Morning routine tasks completed; patient strength, and use of space | During usual care, people with dementia showed slumped posture, sluggish and asymmetric motion, listlessness, minimal awareness of space and physical environment, and poor capacity to complete personal care tasks. Both background music playing and caregiver singing had strong influences on patients’ body and on sensory awareness. Patients had straightened posture, stronger and more symmetric movements, and a greatly increased awareness of themselves and their environment. |
| 6.Gotell et al., 2009 | 9 patients with severe dementia (average age = 84 years) and 5 professional care givers recorded on 27 occasions | Within-subjects design with 3 conditions: usual morning routine, routine+carer singing, and routine+familiar music listening. Qualitative analysis of video recordings. Country: Sweden. Setting: Special care unit for people with severe dementia | Social connection (vocal communication between carers and patients; emotions expressed) | Aggressive behaviour | Compared to no music, the presence of background music and caregiver singing improved the mutuality of the communication between caregivers and patients, creating a joint sense of vitality. Positive emotions were enhanced, and aggressiveness was diminished. Whereas background music increased the sense of playfulness, caregiver singing enhanced the sense of sincerity and intimacy in the interaction. |
| 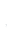7.Kipnis et al., 2016 | N: 159 patients scheduled for surgical procedures (55% women); mean age = 51.5 years (SD = 14.0). 82 assigned to background music (42 classical music and 40 New Age music), 77 in control group. | Mixed design with 3 conditions (Classical music, New Age music, control) x 3 assessment times (evening before surgery, in waiting room, 30 mins later) mixed design. Country: Isreal. Setting: pre-operative ward. | Heart rate,  Systolic BP, Diastolic BP,  O^2^ saturation; Preoperative Anxiety question-naire | Pain (both on 11-item visual analogue scale), | Among pre-operative adults, there was a difference in state anxiety levels between groups such that patients exposed to 30 minutes of New Age music or Classical music showed significantly lower state anxiety than those in the control group. Patients exposed to music showed a decrease in heart rate and SBP and DBP along with an increase in the blood oxygen saturation level. Patients in the control group, by contrast, showed increased heart rate and blood pressure and decreased blood oxygen saturation levels. |
| 8.Nilsson, 2009 | N: 40 (20 in control); Age: average in mid-60s; Sample: Open heart surgery patients | Design: RCT with 2 conditions Music or Control. Quantitative. Country of intervention: Sweden. Setting: Hospital during post-operative care; Intervention length: Single session for 30 mins | Blood gases,  Blood pressure,  Serum oxytocin level | Relaxed behaviour during post-operative period (numeric rating scale) | Compared with controls, patients who listened to 30 mins music during bed rest on their first day following a coronary artery bypass grafting and/or aortic valve replacement under general anesthesia showed significant increases in oxytocin and relaxation. There was no difference in mean arterial blood pressure, heart rate and O2 saturation between the groups. |
| 9. Seinfeld et al., 2016 | N: 40; age >18 years; 68% females | Design: quasi-random allocation to background music (20) or control (20) during virtual reality experience of ascending and descending a tall building (invoking fear of heights) | Heart rate,  Skin conductance, Subjective Units of Distress recorded during the experiment | Post-test recovery | Background music facilitated post-stress recovery in subjects with different degrees of fear of heights, although this anxiolytic effect was only evident in subjective responses taken after the experience, and not in self-reported anxiety scores during the experience or physiological measures. |
| 10.Téllez et al., 2016 | N: 75 women undergoing a breast tissue biopsy; Age: 37-70 years; Sample: no previous biopsy or diagnosis of cancer | Design: RCT with 3 conditions: Hypnosis + music, Music only, or Control (standard care). Quantitative. Country of intervention: Mexico. Setting: Breast Clinic in hospital; Intervention length: single session for 17 mins | Visual analogue scales based on Roth et al. (1998) of stress, anxiety, depression, optimism (pre- and post-biopsy) | Visual analog scales of pain, and general wellbeing | After a breast biopsy, women in the music group reported less anxiety and pain, whereas the hypnosis group showed less anxiety and increased optimism than the control group. |
| 11.Twiss et al., 2006 | N: 86 (44 control) patients in cardiac surgery and surgical intensive care; Age: Over 65 years. | Design: RCT with Music or Control. Quantitative. Country of intervention: USA. Setting: Hospital during post-operative care; Intervention length: during surgery and in the surgical intensive care area | Anxiety (Spielberger STAI) | Length of intubation time (minutes from time patient left operating room until they were extubated) | Older adults in the music listening condition had significantly less anxiety than the control group. Music listening reduced the length of intubation time following CABG and valvular surgery in patients who listened to music than those who did not listen to music. |
|  |  |  |  |  |  |
|  |  |  |  |  |  |
| Intentional music listening |  |  |  |  |  |
| 12.Clark, et al., 2016 | N: 27 cardiac rehabilitation patients (21 men, 6 women); mean age = 67.3 (SD = 4.3) recruited immediately after discharge for a program using music during walking exercise. | Design: Qualitative RCT with Music listening or Control (usual care) over 6 months.  Country: Australia. Two phase study: 1. participants preferred music was assessed using Grocke's (1999) SMMA; 2. interviews immediately post intervention to assess participant's experiences of using individualized music listening to support walking exercise. | Physical and physiological arousal during walking | Exercise adherence (6 months post discharge) | For older adults with cardiac disease, a holistic approach to music selection considering general well-being and adjustment issues, rather than just exercise performance, may improve long-term lifestyle changes and adherence to physical activity guidelines. Music listening promoted physiological arousal during exercise. |
| 13.Drzymalski et al., 2017 | N: 99 women in labor preparing for an epidural catheter. Mean age music group 31 years (SD = 6); control group 32 years (SD = 5) | Design: RCT with Music (listening to patient’s preferred music on a Pandora® station broadcast through a speaker) or Control (no music).  Country: USA.  Duration of intervention: average of 31.1 minutes (SD = 7.67). | 3 measures of patients’ anxiety: 0-10 analogue rating, calm rating,  Two questions from STAI and anesthesia provider anxiety. | Pain, patient satisfaction. | Laboring women listening to preferred music during epidural catheter placement reported higher post-procedure anxiety and no improvement in pain or patient satisfaction. No differences were found between conditions in anesthesia provider relaxation or calmness during epidural catheter placement. |
| 14.Fernando et al., 2019 | N: 24, 12 men, 12 women. Mean age 53.33 years (SD = 10.11). Adult resident patients suffering chronic pain at a tertiary cancer care institution. | Design: Randomized Crossover Trial.  Country: Sri Lanka.  Intervention: Patients were monitored for selected outcomes without music on day 1, and then monitored with music listening (day 2: 28-minute music clip). Measured over 24 hours after music exposure. | Pulse rate,  Respiration rate,  Systolic blood pressure,  Diastolic blood pressure,  pupillary size. | Pain, on visual analog scales augmented with Wong Baker Faces Pain Scale. | Compared to a day without music listening, a day with a period of listening to culturally familiar instrumental classical music significantly alleviated pain, anxiety, and mood in cancer patients undergoing treatment. |
| 15.Finlay et al., 2016 | N: 98 patients (40 males) scheduled for primary knee replacement surgery. Age males M = 68 years, SD = 7.96; females M = 68 years, SD = 8.14. | Design: Between groups with 5 experimental groups: silent control or four types of music-listening.  Country: Scotland.  Setting: 15-minute intervention per day of inpatient stay. | Salivary cortisol,  Mood | Pain intensity (Visual and Numerical Rating Scales); Short-Form McGill Pain Questionnaire (SFMPQ; Melzack, 1983), pain interference (Brief Pain Inventory (BPI Short-form; Cleeland, 1992). | Salivary cortisol concentrations showed that music with high harmonicity/rhythmicity reduced cortisol concentration to a greater extent on Day 1 than music with low harmonicity/rhythmicity. Knee replacement surgery patients showed reductions in pain from pre- to post-test, indicating silent relaxation was as effective as music-listening. |
|  |  |  |  |  |  |
| 16.Guétin et al., 2016 | N: 35 (17 women, 18 men); mean age 61.26 ± 11.64 years, prior to undergoing a coronary artery angiography. | Design: uncontrolled, pre-post.  Country of intervention: New Caledonia.  Setting: Hospital.  Intervention: Participants used a phone app with choice of 20 types of music. Measures taken at a single time-point at pre- and post- 20 minutes music listening. | Anxiety (11-item visual analogue scale) | Pain (11-item visual analogue scale), satisfaction (four categorical responses) | In coronary artery angiography patients, there was a significant reduction in anxiety after listening to chosen music. No reduction of self-reported pain, although only a few participants indicated pain. No gender differences were found. |
| 17.Helsing et al., 2016 | N: 41 community dwelling women (21 in relaxation + music listening group, 20 in relaxation only control). Mean age 32 years. | Design: RCT  Intervention: experimental group listening to preferred music on mp3-players daily for 30 minutes during a two-week period. One week they listened to their own chosen relaxing music and the other their own chosen energizing music. Control group were asked to relax for 30 mins a day in their own home.  Country: Sweden.  Setting: Own homes | Emotions (a short version of Positive Affect Negative Affect Scale),  Stress (Perceived Stress Scale),  Salivary cortisol | Health (Symptoms of Illness Checklist). | Women who listened to their own chosen music reported significantly higher intensity positive emotions and less stress than when they relaxed without music. It did not matter whether the music was relaxing or energizing. There was also a significant decrease in cortisol from the baseline week to the second music intervention week. The control group’s reported stress levels, perceived emotions and cortisol levels remained stable during all three weeks of the study. Symptoms of Illness Checklist scores decreased over time in both groups. Listening to preferred music may be a more effective way of reducing stress and cortisol levels and increasing positive emotions than relaxing without music, however, illness symptoms can be reduced with relaxation practice regardless of music listening. |
| 18.Hides et al., 2019 | 169 young people (mean age = 19.9 years, SD = 2.5; 79% females) with at least mild distress (Kessler 10 score >17) using Music eScape phone app for emotion regulation | Design: RCT  Intervention: 2 conditions (1) immediate app use 4 weeks or (2) 4 weeks wait list. Participants were assessed at five time-points. | Emotion regulation skills (DERS-SF) | Wellbeing (Mental Health Continuum-short form), distress (K10) | In this sample of young people, no significant differences were found between the immediate and delayed music app use groups on emotion regulation, distress, or well-being found at 1 month. Both groups achieved significant improvements in 5 of the 6 emotion regulation skills, mental distress, and well-being at 2, 3, and 6 months. |
| 19.Ihara et al., 2018 | N: 51 adults with dementia scoring ≤24 on the MMSE. Intervention group mean age = 81.17 years (61% female); control group mean age = 83.79 years, (75% female). | Quasi experimental design  Intervention: two conditions (1) Music and Memory program of personalized playlists twice weekly for 6 weeks or (2) wait list control. Observations and assessments at pre-, post- and 6 weeks post intervention.  Country: USA.  Setting: Adult Day Health Care Centers for people with dementia. | Observations: connecting to music and engaging socially; Mood (Cornell Scale for Depression in Dementia) | Agitated behaviour (Cohen-Mansfield Agitation Inventory) | There were no significant results on standardized measures, however the behavioral observations showed a positive change in mood and a decrease in agitation in individuals living with dementia attending Adult Day Health Centers. From pre- to post-intervention, there were significant increases in joy, eye contact, eye movement, being engaged and talkativeness, and a decrease in sleeping and moving or dancing. |
| 20.Kulibert et al., 2019 | N: 24 people 58% men) with mild to moderate dementia and 24 family carers. | \| Design: single arm pre- to 3 months post. Quantitative and qualitative. Country: USA. Setting: participants' homes. Participants were given iPods with playlists chosen after a music interview (Music & Memory process). \| \| --- \| | Autobiographical memories stimulated through personally relevant music listening. | BASQID (Trigg, et al., 2007) measures health and quality of life in people with mild to moderate dementia; Caregiving Distress Scale; The Revised Memory and Behavior Problems Checklist; GAIN. | In these people with dementia, there were no significant effects of selected music listening in the quantitative measures, possibly due to low sample size after attrition. However, qualitative responses indicated participant enjoyment and relaxation to music; carers reported some respite. Music listening was not always shared (it depended if through headphones or speakers). |
| 21.Mercadie et al., 2015 | N: 22 (100% women, mean age: 51.4, SD = 10.4 years) with Fibromyalgia, recruited from the public. | Between groups design: 4-week study in which patients with fibromyalgia listened to either preselected musical pieces or environmental sounds when they experienced pain in active (while carrying out a physical activity) or passive (at rest) situations. Country of intervention: France. Setting: in community. | Emotional effects of listening to music (or environmental sounds) | Pain and fatigue measured on 10-point visual analogue scales, before, after, and 10 minutes after listening | When patients with Fibromyalgia listened to 20 minutes of music or environmental sounds at rest, pain and fatigue levels were significantly reduced after listening, with no difference of effect between the two sound stimuli. This effect lasted 10 minutes after the end of the listening session. In active situations, pain did not increase in presence of the two stimuli. |
| 22.O'Callaghan et al., 2012 | N: 100 patients commencing radiotherapy for cancers (59% men), mean age: 58 years (SD = 12.7) controls and 57 years (SD=14.2) music condition. | Design: random assignment to music listening or control (treatment as usual). Quantitative and qualitative methods. Country of intervention: Australia. Setting: hospital. Music condition participants were asked to bring their own music to their radiotherapy treatment. | ; Anxiety (STAI) | Radiotherapy experience questionnaire - feeling supported, distracted or that treatment time seemed faster. | During initial radiotherapy, patients’ state anxiety scores did not reduce when they listened to preferred music. However, those who received music were more likely to want music in future sessions (89% v 60% of controls). Qualitative responses indicated that music listening distracted them from the hospital noises and procedures and helped pass the time more quickly. |
| 23.Särkämö et al., 2008 | N: 60 patients in acute recovery from middle cerebral artery stroke. | Design: randomized controlled with 3 conditions: listening (minimum 1 hour per day for 2 months) to favorite music from CDs, listening to audiobooks, or usual treatment control. Measures taken at baseline, 3 and 6 months. Country of intervention: Finland. | Mood states (POMS) | Neurocognitive performance on tests of verbal memory, short-term and working memory, language, visuospatial cognition, music cognition, executive functions, focused attention and sustained attention. Stroke and Aphasia Quality of Life Scale-39 | Stroke patients showed significant group x time interactions on verbal memory and focused attention: verbal memory was better in the music listeners than the audiobooks or usual treatment controls. Focused attention was better and there was less depressed and confused mood in the music group than the control group. Listening diaries kept by the music group patients showed that 62% of all music selections were popular music (pop, rock or rhythm and blues), 10% was jazz, 8% was folk music and 20% was classical or spiritual music. No significant group differences in QOL at the 3 or 6 months follow up. |
| 24.Sorensen et al., 2019 | N: 78 adults leading a 'busy and stressful life' were recruited from the community (mean age = 40.15; SD = 13.68); 90% women. | Mixed design with random assignment to 3 conditions: Convergence (composed guitar music+loving kindness meditation); LKM only or Guitar Music only. 3 assessments: pre-program, post-program, and 4-week follow-up. Intervention was a single 2-hour workshop with 2 clinical psychologists and recordings that participants could take home to practice. Country: Australia. Setting: university classroom. | Mindfulness (FFMQ),  Compassion (FSC, SCS) | Warwick Edinburgh Mental Wellbeing Scale; and K6 psychological distress | For this sample of stressed community dwelling adults, music meditation, meditation only, and music only programs produced significant effects for mindfulness, wellbeing, and psychological distress (K6). There were no group x time interactions, indicating that all three interventions produced similar effects. |
| ***Music Sharing*** |  |  |  |  |  |
| 25.Clements-Cortes, 2017 | N: between 600-800 (varied depending on concert) adults with and without dementia living in care facilities; Age: 55+ years | Design: pre-post; Qualitative and quantitative. Country of intervention: Canada. Setting: Chamber music concert; Intervention length: 6 months, 30 live concerts of 4 types of chamber music | Engagement, enjoyment, and special moments with others; mood, social connection and meaning. | Pain, and energy. | For these older adults attending live chamber music concerts, pain was reduced, and energy and mood improved. The survey and observation data demonstrated that when attending the concerts, older adults experienced: engagement, enjoyment, and special moments with others, and connection and meaning were fostered. |
| 26.Shibazaki & Marshall, 2017 | N: 53 aged 71-97 years; 27 dementia clients (UK-16/JP-11), 13 family members (UK-8/JP-5), 9 members of nursing/volunteer staff and four care/activities managers (UK-5/JP-4). | Design: interviews with qualitative analysis; Countries of intervention: England & Japan; Setting: Concert halls; Intervention length: Single session for 1 hour | Identity & communication (lucidly, vocality), agency (music requests) | Agitation, antisocial behavior, wellbeing | Clients with mild to mid-stage dementia noted increased levels of cooperation, interaction, and conversation. Those with more advanced dementia showed decreased levels of agitation and anti-social behavior. Staff members reported increased levels of care, cooperation, and opportunities for assessment. Family members noted an increase in the levels of well-being in their partner/parent as well as in themselves. Family members reported that clients often became more lucid, vocal, and animated with an increased motivation to talk and interact, not only with members of the family but with other residents. Music was often an integral part of an individual’s identity, with clear preferences, individuals made music requests linked to life events or to individual people. |
| 27.Toccafondi et al., 2017 | N: 242 cancer patients attending the Music Givers program, 95 men (139 Control; 103 intervention); Age: 18-90 years. | Design: quasi experimental (it was recorded whether the patient attended a music event during their admission to hospital). Country of intervention: Italy; Setting: Live music performance & buffet/socialisation with musicians in a hospital; Intervention length: One event per week for 2 hours (45-60 min music) | Social connections among patients, staff, and musicians; Mood (Hospital Anxiety and Depression Scale). | Multidimensional well-being scale; distress thermometer; visual analogue scales (score range = 1–10) to assess pain, fatigue, and five areas of well-being | Compared to the control group, cancer patients who attended the Music Givers program showed less distress at discharge according to the DT, lower HADS–Anxiety and HADS–Depression scores, and higher scores on all the well-being scales, except spiritual well-being. No between-group differences were found in terms of pain and fatigue scores at discharge. |
| 28.Travers & Bartlett, 2011 | N: 154; Age: 60+ years; Sample: 67 community-dwelling persons and 46 residents of residential care facilities listening to the Silver Memories radio program daily | Design: pre-post. Country of intervention: Australia. Setting: Radio program in participants own homes; Intervention length: once per day for 3 months | Social connection/loneliness; Mood (depression) | Quality of Life | Older adults who listened to Silver Memories radio program daily showed significant improvement in depression and QOL from baseline to follow-up but there was no change on the measure of loneliness. The results did not vary by living situation (community vs. residential care), whether the participant was lonely or not lonely, socially isolated or not, or whether there had been any important changes in the participant’s health or social circumstances throughout the evaluation.  It appears that listening to Silver Memories improves the QOL and mood of older people although there was no evidence to support a social mechanism for this. |
| 29.van der Vleuten et al., 2012 | N: 45 adults with mild to severe dementia, 15 men; Age: not detailed. | Design: cross sectional, observational. Quantitative. Country of intervention: the Netherlands; Setting: Aged care home; Intervention length: Live music performance, single session for 45mins | Participation, social connection, positive and negative emotions | Wellbeing, and relationship quality in care relationship | According to carers’ ratings made at the end of the concerts, live music had a positive effect on human contact, positive emotions and negative emotions, especially for the mild dementia group and an improved relationship between caregiver and receiver. |
| ***Instrument Playing*** |  |  |  |  |  |
| 30. Bugos & Kochar, 2017 | N: 34 older adults, mean age = 70.79 years (*SD*= 6.20), 18% males; with little to no musical training | Design: within-subjects with three time points: pre-training, pre-training two (after baseline phase), and post-training. Country of intervention: USA; Setting: piano studio. Intervention: intensive piano lessons. 30 hours focused music theory, finger dexterity exercises, bimanual coordination exercises, technical exercises, performance duets, standard piano repertoire | Cognitive and motor skill stimulation | Cognitive health (performance on neuropsychological tests) | After piano training, participants showed significantly better verbal fluency and processing speed however, no difference was found in verbal memory performance. The authors concluded that short-term intense music instrument programs may be an appropriate structure for a cognitive training program with the added benefit of increased interest in continued music participation. |
| 31.Bugos et al., 2007 | N: 39 healthy older adults (aged 60–85 years) recruited from community for individual piano lessons to enhance cognitive health. 21 experimental group (4 males, 17 females); 18 in the control group (4 males, 14 females) | Design: controlled trial with 2 conditions. Intervention group received 20 sessions basic piano technique, dexterity exercises, piano literature, music theory; piano repertoire (Alfred Basic All-in-One Method); Control group received 20 sessions music listening.  Country: USA | Musical ability measured over time (Advanced Measures of Music Audiation), motor exercises | Cognitive health (neuropsychological test performance) | 6 months after starting twice weekly piano lessons, healthy older adults showed significant improvements on the trail making test and digit symbol measures compared with the controls who did music listening. Individualized piano instruction may be an effective intervention against age-related cognitive decline. |
| 32.Knapp & Silva, 2019 | N: 28 (12 band members and 16 non-member residents of a homeless shelter); also 5 members and 2 employees were interviewed. | Design: 3 years longitudinal; quantitative (compared scores on Interpersonal Support Evaluation List between band members and non-member shelter residents) and qualitative methods. Country: USA. Setting: homeless shelter. | Perceived social support and self esteem | Social determinants of health: housing status, criminal behaviour. | In these residents of a homeless shelter, qualitative data indicated that members of the band had improved perceptions of social support and self-esteem and some managed to end their homelessness. They reported reduced recidivism and improved case management. Quantitative analyses showed no statistically significant differences in ISEL between music band members and non-member residents for social support, belonging support and self-esteem support. |
| 33.Mansens et al., 2018 | N: 1101 (578 females, 523 males) aged 64 yrs and older; 137 (11.5%) only sang; 62 (5.2%) only played a musical instrument; 59 (5%) did both. 824 did not make music; 19 (1.7%) did not specify type of music made. | Design: group comparison of instrumental music making, singing, controls; cross sectional survey. Country: The Netherlands. | Time spent making music (music making and singing measured on the LASA Physical Activity Questionnaire) | Cognitive health (performance on neuropsychological tests) | In community dwelling older adults, regular music making was associated with improved letter fluency, learning, attention/short term memory. No significant findings for time spent making music. Music playing resulted in higher processing speeds compared to singing. |
|  |  |  |  |  |  |
| 34. Perkins & Williamon, 2014 | N: Study 1 = 98 older adults (74 females; 22 males; 2 unknown); mean age = 67.87 years (*SD* = 8.76). Study 2 = subgroup of 21. Evaluation of the Rhythm for Life project at the Royal College of Music, UK. | Design: controlled, with 30 in a comparison group (U3A Shared Learning project) and 38 in the music making group. 10-week program of music making (keyboard, guitar, recorder or djembe drum), taught by a RCM student either individually or in groups; 1/wk for 60 minutes per lesson. Study 2: same as above but utilizing 2 students as teachers; groups of 3-8 participants; community venue; 60 minutes weekly. | Mechanisms: mood (pleasure), social interactions, engagement in day-to-day life, achievement | Study 1: health promoting behaviours; Wellbeing (Short-WEMWBS). | Study 1: Learning in older adulthood offers significant benefits to wellbeing, with music enhancing health promoting behaviors (especially interpersonal relations and physical activity). Study 2: learning music can enhance wellbeing through 6 mechanisms. |
| 35. Ritchie & Williamon, 2011 | N: 404 primary school children (200 boys; 204 girls); 138 completed re-test at 9 months | Design: cross sectional survey to examine associations between time spent listening to music, reading, participation in extra-curricular activities (e.g., sports, music lessons, dance); self-efficacy and wellbeing in primary school children. Country: UK. | Self-efficacy | Well-being; Strengths and Difficulties questionnaire | Primary school children learning a musical instrument or singing had significantly higher self-efficacy scores than children who were not. Girls’ scores were higher than boys’ scores. Time spent listening to music, participation in individual sports, dancing, doing homework and reading for pleasure correlated positively with self-efficacy for music learning scores. There was a correlation between self-efficacy and well-being. Self-efficacy correlated negatively with hyperactivity, emotional symptoms, and conduct problems (SDQ). |
|  |  |  |  |  |  |
| 36.Seinfeld et al., 2013 | N: 29 adults aged 60–84 years. 13 in Piano group (9 females,4 males), 16 in Control (13 females, 3 males). | Design: two groups, pre-post. The intervention group attended weekly group piano lessons, lasting 90 minutes, over 4 months; the control group engaged in other leisure activities (e.g., physical exercise)  Country: Spain. Setting: community center | Cognitive stimulation of new learning; Mood (Beck Depression Inventory; Profile of Mood States). | Cognitive health (neuropsychological test performance), wellbeing and quality of life | The older adults in the piano training group significantly improved on measures of executive function, inhibitory control, and divided attention. There was a trend indicating an enhancement of visual scanning and motor ability was also found (Trial Making Test, part A). Piano lessons decreased depression, induced positive mood states, and improved the psychological and physical quality of life. |
| 37.Vik et al., 2018 | N: 30, 8 weeks of piano lessons for 3 groups: 7 patients with mild traumatic brain injury (4 male, 3 female, mean age 38 yrs); Control group with music training (5 male, 6 female, mean age 33 yrs); Control group without music training (4 male, 7 female, mean age 33 yrs) | Design: 3 groups x 2 times (pre- and post- the 8 weeks program); cognitive tests, fMRI, and qualitative analysis of interviews;  Country: Norway.  Setting: University hospital | Functional MRI; music pitch discrimination task; tonika-dominant-tonika task. | Cognitive health (performance on neuropsychological tests) | In patients with mild traumatic brain injury. neuropsychological tests pre–post music intervention demonstrated significant enhancement of cognitive performance in both Group 1 and Group 2. The results of the CVLT clearly indicated an effect of musical training on cognitive performance in Group 1 and Group 2, which was absent in the control group. Group 1 had a post-intervention score that was at a comparable level as the baseline of Group 2 and Group 3. Hence, the performance improved up to the normal level. Qualitative data from interviews pre–post in Group 1 supported these results. |
| 38.Wilson & Macdonald, 2019 | N: 37 adults (12 females, 25 males) with mild to profound learning disabilities; level of independence ranged from completely independent, living alone in supported accommodation, to needing one-on-one care | Design: descriptive interview study, qualitative analysis.  Intervention: 10 weekly community music workshops.  Country: Scotland. | Enjoyment, social participation, improved mood, self-esteem. | Disability inclusion and wellbeing | People with disabilities experienced barriers to participation such as: limited access to enjoying or learning music; boredom; isolation; limited networks; lack of experience in new social contexts; lack of confidence; low mood or self-esteem. The benefits of their participation included enjoyment and enthusiasm to engage in music; increased self-confidence; being happier and more relaxed; better able to deal with unfamiliar situations and people; better participation in social activities for longer periods of time. |
| ***Group Singing*** |  |  |  |  |  |
| 39.Beagan & Etowa, 2011 | N: 50 women; Age: 40-60 years; Sample: African Canadian women living in Nova Scotia, Canada. Most were members of a Christian Church | Design: semi structured interviews with qualitative analysis. Country of intervention: Canada. Setting: Church. | Spiritual, cultural identity, social connection | Mental and physical health | For these minority African Canadian women living in Nova Scotia, choir singing and listening to spiritual music helped protect against the psychological effects of racism. The women described how singing supported their physical and mental health through a spiritual connection with the Lord and through their cultural connection with the African Christian community. |
| 40.Dingle et al., 2013 | N: 21, 9 men; Aged: 31-74 years; Sample: Marginalized adults with chronic mental health problems | Design: longitudinal, 3 interviews (beginning, 6 months, and 12 months) with qualitative analysis Country of intervention: Australia. Setting: Community hall. Intervention length: Weekly for 12 months | Social connection, choir identity, motivation, emotion regulation, self esteem | Mental health, physical health, social determinants (employment). | For these marginalized adults, weekly choir singing was associated with mental health benefits and improved general functioning over time. A range of mechanisms were revealed including social identity (as a choir member), emotion regulation, motivation, self-esteem, achievement. |
| 41.Dingle et al., 2020 | N: 46, 81% female; Age: M = 81 years; residents of a retirement village. | Design: RCT with Music Program or Wait list control, and two time points (pre and post). Country of intervention: Australia. Setting: Community hall. Live Wires program: weekly 2 hour sessions comprising cognitively challenging warm up exercises, group singing, songwriting, and afternoon tea, over 8 weeks. | Social identification with the Live Wires group and with the retirement village | Well-being (Warwick-Edinburgh Mental Well-being Scale) and cognitive health (Addenbrooke's Cognitive Examination III) | In these healthy retirement village residents, participating in the Live Wires program was associated with improved cognitive performance on the ACE-III and increased identification with the retirement village, while the wait listed control participants did not show these benefits. There was no condition x time interaction for wellbeing scores, although they were high in both groups. Qualitative analysis of interviews revealed further evidence of the social, cognitive and wellbeing benefits of participating in Live Wires. Some negatives were also noted such as difficulties working as a group in the song writing process, difficulty with the warm-up exercises, difficulty making time for weekly music with existing commitments. |
| 42.Fu et al., 2018 | N: 49 (10 men); residents of three senior living communities in the Pacific Northwest of the US. Age: 60+ years; Sample: Primarily Caucasian | Design: pre- post, quantitative.  Country of intervention: United States. Setting: Retirement communities; Intervention: 75 min session weekly for 12 weeks of pre-singing exercises, song-singing and learning, and socialization. | Physical activation and motivation; Control, Autonomy, Pleasure and Self-Realization (CASP-19). | Cognitive health (performance on neuropsychological tests), lung health (spirometry & respiratory pressure meter, oximeter), Quality of Life | In these retirement village residents, participating in weekly breathing exercises, group singing, and socializing was associated with significant improvements in cognitive health (e.g., verbal fluency and immediate word recall) and lung health. Program satisfaction was rated an average of 9 out of 10. There was no significant change in quality of life. |
| 43.Johnson et al., 2020 | N: 390, 35% men (208 in choir and 182 in wait-list control); Age: 59-93 years (M = 71y); Sample: Fluent in either English or Spanish, over half non-Latinx Black, Asian, or Latinx. | Design: cluster randomized controlled with 2 conditions (intervention and wait list control). Country of intervention: United States. Setting: Administration-on-Aging-supported senior centers; Intervention: Community of Voices choirs - 90 min sessions weekly for 6 months. | Loneliness, interest in life, physical and motor effects (Standing Balance measure & gait speed) | Patient Health Questionnaire (PHQ); cognitive health (neuropsychological tests); health care costs. | Compared to controls, choir members experienced significantly greater improvements in loneliness and interest in life. However, no significant group differences were found for cognitive or physical outcomes or for health care costs. |
| 44.Lamont et al., 2018 | N: 42 (5 men); Age: 55-82 years; Sample: Adults in the community | Design: series of interviews, world café style focus groups with qualitative analysis. Country of intervention: England; Setting: Community hall; Intervention length: Weekly for 4 years | Social relationships, meaning and accomplishment, mood | Wellbeing benefits, healthy ageing | Positive emotions were found to be fundamental to the enjoyment of the experience, achieved engagement and the challenge of singing; connections to others through social interactions within and beyond the choir; sense of meaning alongside the accomplishment of learning and performing. Some reports of flat or negative mood following the pleasure of rehearsals. |
| 45.McNaughton et al., 2016 | N: 23 (10 men); Age: 51-91 years; Sample: Diagnosed with COPD or interstitial lung disease | Design: interviews and focus groups, qualitative analysis.  Country of intervention: New Zealand; Setting: Community hall; Intervention length: Weekly for 6-9 months | Social connections and a shared purpose were key, as well as physical activation | Health benefits included improvements in breathing, sputum clearance and exercise tolerance, and improved well-being. | This analysis of interviews with members of the Sing your Lungs Out choir revealed four important functions of singing in the choir: being in the ‘right space’, connection, purpose and growth, and participation in a meaningful physical activity. |
| 46.Osman et al., 2016 | N: 20; Age: 51-91 years; Sample: White, British adults. 10 with dementia and 10 carer/family members | Design: interviews with qualitative analysis.  Country of intervention: England; Setting: Community hall; Intervention length: Weekly for 2 months | Social inclusion and support, shared experience, improvements in relationships, memory, and mood | Wellbeing: lifting the spirits and acceptance of the diagnosis (dementia) | This analysis indicates that Singing for the Brain^TM^ choir participation improved wellbeing for people with dementia and their family members / carers through social, mood and memory mechanisms. |
| 47.Pearce et al., 2016 | N: 135 (17 men), 84 in singing groups, 51 other groups (creative writing and craft); Age: 18-83 years (*M* = 56 years); Sample: community dwelling adults, primarily Caucasian. | Design: repeated measures with 3 time points over 7 months, comparison of singing groups with non-singing groups (creative writing/craft).  Country of intervention: England; Setting: Community-based adult education classes; Intervention length: Weekly for 7 months | Social bonding, mood (anxiety and depression) | MOS 36-SF of the RAND Health-related Quality of Life scale for physical health. Flourishing and Satisfaction with Life scale (SWLS) for well-being. | This study found that adults attending community groups experienced better physical and mental health and satisfaction with life over time in both the singing and non-singing groups (creative writing, crafting). Higher collective-bonding at timepoint 3 significantly predicted increased flourishing, reduced anxiety, and improved physical health independently of baseline levels. In contrast, individual relational bonding showed no such effects, suggesting that it is feeling part of a group that particularly yields health and well-being benefits. |
| 48.Perkins et al., 2018 | N: 54 mothers of infants up to 40 weeks of age experiencing postnatal depression (37 in singing condition, 17 in group creative play control); Age: 22-45 years, British,  French, Polish, Canadian, Columbian, Australian, Japanese,  Italian and North American nationalities. | Design: RCT, semi structured focus groups with qualitative analysis.  Country of intervention: England; Setting: Community. Intervention length: 10 weeks | Socially and culturally inclusive groups; creative experience; sense of achievement and identity; enhanced bond with infant | Mental health (decreased postnatal depression) | This study found that new mothers experiencing symptoms of postnatal depression benefited from involvement in both the singing and creative play groups. The group activities acted on their mood via several mechanisms: (1) a shared experience for mothers of seeing babies develop and enjoy a creative activity; (2) learning new activities to do with babies, and increased confidence in doing this outside of the intervention sessions themselves; (3) an enhanced sense of ‘feeling good’; (4) a sense of group belonging, in which knowledge about motherhood can be shared; (5) a sense of structure and routine in daily life; (6) a calm and inclusive environment, facilitated by high quality creative leaders and support team. There was little evidence that group singing was more effective than group creative play. |
| 49.Särkämö et al., 2013 | N: 89 dyads, 24 men; Age: 60+ years (*M* = 79 years); Sample: Finnish speaking people with mild-moderate dementia in patient-caregiver dyads. | Design: RCT with dyads randomized  to a singing group, a music listening group, and a usual care control group. Assessments were made a baseline and 2 follow ups over  9 months. Country of intervention: Finland; Setting: Day activity centers, inpatient centers; Intervention length: Weekly for 12 weeks. | Cognitive effects, mood effects. | Wellbeing of people with dementia (Cornell-Brown Scale for QoL & QoL for Alzheimer's Disease scale) and their carers (GHQ-12 & Zarit Burden Interview for caregivers). | Compared with usual care, both singing and music listening improved mood, orientation, and remote episodic memory and to a lesser extent, attention and executive function and general cognition. Singing also enhanced short-term and working memory and caregiver wellbeing, whereas music listening had a positive effect on QoL. |
| 50.Stewart & Lonsdale, 2016 | N: 375 (53% females) recruited from the community by email and social media; Age: 18-78 y, M = 37. Of these, 125 choral singers, 125 solo singers and 125 team sport players. | Survey study with a between groups design: choir singers v solo singers v team sports players all amateur level. Country of intervention: England; Setting: Community hall; Intervention length: Weekly for 2 months. | Socialgroup entitativity, Self- Regulation (SRQ-E), Autonomy, Competency and Relatedness | Hedonic wellbeing; Warwick Edinburgh Mental Health and Wellbeing scale; Satisfaction with Life. | Choral singers and team sport players reported significantly higher scores on one measure of subjective well-being than solo singers, while no significant differences were found between team sport players and choral singers on the same scale. In relation to process variables, choral singers considered their choirs to be significantly more entitative than team sports players considered their teams, regardless of group size. Autonomy was higher for solo singers than for both team sport players and choral singers, and significantly higher for team sport players than for choral singers. Team sport players were also found to report significantly higher self regulation scores than both choral singers and solo singers, but no significant  differences were found between choral singers and solo singers. This study provides preliminary evidence that wellbeing effects of different group activities may occur via different psychosocial processes. |
| 51.Weinstein et al., 2016 | N: 124 in large choir (18 men), 20-80 in small choirs (22 men); Age: 21-75 years; Sample: Community members, primarily Caucasian. | Design: pre-post a 90- minute singing session; comparison of smaller and large choir. Country of intervention: England; Setting: Community choirs in Greater London area; Intervention length: single choir rehearsal session | Positive & negative affect (PANAS short form), social inclusion and social connectiveness | Pain thresholds (as a proxy for endorphin release) | Feelings of inclusion, connectivity, positive affect, and pain threshold (proxy for endorphin release) all increased across singing rehearsals. The influence of group singing was comparable for pain thresholds in the large versus small groups. Levels of social closeness were found to be greater at pre- and post-levels for the small choir condition. However, the large choir condition experienced a greater change in social closeness as compared to the small condition. |
| 52. Williams et al., 2019 | N: 59 (34 choir members and 25 creative writing group members); marginalized adults with mental health conditions, mean age = 46 years, 51% females. | Design: one-year prospective study with 3 assessment points. Country: Australia. Setting: Community‐based and facilitated by arts professionals. | Identification with the choir or creative writing group | Wellbeing (Warwick Edinburgh Mental Wellbeing Scale) | Multilevel modelling analyses demonstrated that participants’ mental wellbeing significantly improved over time. Greater identification with their arts‐based group was significantly related to an increased rate of improvement in mental wellbeing. The  trajectory of improvement in mental wellbeing did not differ between participants engaged in the choir or creative writing group. |
| ***Movement and Dance*** |  |  |  |  |  |
| 53. Doi et al., 2017 | N: 201 older adults with mild cognitive impairment living in the community, 48% men. Of these, 67 in dance condition; 67 music instrument condition; 67 control (health education); Age: 70+ years (M = 76 years) | Design: randomized with 3 conditions: dance, music instrument, or control; two assessments (pre- and post- the 40-week intervention). Country of intervention: Japan; Setting: Community hall; Intervention length: 60 min weekly session for 40 weeks | Cognitive stimulation and physical activity (steps per day). | Cognitive health (neuropsychological test performance) | At 40 weeks, older adults with mild cognitive decline who attended a dance group showed better story memory recall scores than those in the health education control condition whereas the music instrument group did not show this benefit. There was no significant improvement in word list recall in any of the conditions.  Both dance and music instrument groups showed improved general cognitive functioning (MMSE scores) compared with controls. There was no difference between the 3 conditions in the non-memory cognitive tests or physical activity levels. |
| 54. Jeong & Kim, 2007 | N: 33 (23 men) recovering from stroke at least 6 months prior. 16 in movement condition, 17 in control (received  information about available usual care services); Age: M = 60 years; Sample: Had poor to moderate muscle strength, and disability on one side of the body | Design: two group randomized trial.  Country of intervention: South Korea. Setting: Neighborhood community health center. Intervention: rhythmic auditory stimulation (RAS) informed music movement program, 2 hours per week for 8 weeks | Motor flexibility, mood (Profile of Mood States), perceived Relationship quality. | Quality of Life | Participants in the music movement group gained a wider range of motion and flexibility, had more positive moods, and reported increased frequency and quality of interpersonal relationships than those in the control condition. Both conditions reported an increased quality of life over the 8 weeks, so there was no significant group x time interaction. |
| 55.Murrock & Gary, 2010 | N: 126 African American women. 66 in the dance condition, 60 in control – usual activities and health information. Age: *M* = 36-82 years; Sample: women recruited from two Baptist churches who were sedentary and obese | Design: quasi experimental with 2 conditions (dance or control) and 3 assessment points (baseline, 8 weeks and 18 weeks). Country of intervention: USA; Setting: Community hall; Intervention: culturally specific dance program twice per week for 45min sessions over 8-weeks | Physical activity (Physical Activity Scale for the Elderly) | Healthy weight indicators (BMI, body fat %) | There were no differences between the two conditions at baseline in age, comorbid health conditions, weight, body fat, BMI, marital status, SES, or education. The culturally specific dance intervention significantly decreased body fat and BMI in sedentary African American women from baseline to 8 weeks and was maintained at 18 weeks when compared to the participants who did not receive the intervention. |
| 56.Vlismas et al., 2013 | N: 46, 24 mother-infant dyads in Ex 1 (Age M_Mothers_ = 33y, M_Infants_ = 3.2 months) and 22 mother-infant dyads in Ex 2; (Age M_Mothers_ = 31y, M_Infants_ = 3.3 months). Sample: Healthy first-time mothers and their infants who spoke English and had a score <10 on the Edinburgh Postnatal Depression Scale | Design: randomized to one of 4 conditions: (i) M&M-F2F, (ii) M&M-Only, (iii) F2F-Only and (iv) No M&M-No F2F (control group).  Country of intervention: Australia; Setting: Experiment 1: 8-10 group of mother infant pairs (location not stated) or at home for individual M&M group. Experiment 2: own homes; Intervention length: 5 weeks for 90 mins (60 mins intervention, 30 mins morning tea) | Social bonding and attachment with infants; mood effects. | Mental health prevention in new mothers. | The results of these two experiments show that the 5 weeks Music and Movement program delivered face to face or in audio recorded form for home use was associated with increases in the mothers’ self-reported use of music and enjoyment of interactions with their infants; attachment to their infants; increases in dyadic reciprocity between mother and infant and increases in attentional and affective, but not the didactic instructional, aspects of mothers’ speech. |
| ***Lyrics and Rapping*** |  |  |  |  |  |
| 57. Peterson et al., 2008 | N: 126 university students (70 female, 56 male); Age: M = 19.53 years, 89% Catholic | Study design: within subjects experimental (pre- post music listening). Country: USA. Setting: university. Participants were given a CD player and headphones, and the lyric sheets so that they could read the liner notes with the music. The three songs lasted about 20 minutes. | Personality traits; self- esteem; affective states | Mental health (suicide ideation in story-writing) | This study examined the potential effect of listener individual differences and listening to music with nihilistic lyrics on mood and suicide-related story-writing post listening. Low openness to experience, high neuroticism and low self-esteem predicted higher levels of suicide-related content in projective story-writing, as did knowing a suicide victim. Personality measures and post-listening mood predicted remembering many nihilistic lyrics. Individual differences were modestly associated with suicidal thoughts after listening to the music. |
| 58. Simmons-Stern et al., 2012 | N: 29 older adults, 12 with probable Alzheimer’s Disease (Age M = 81.17 years, 6 males, 6 females), 17 healthy older adults (Age M = 78.63 years, 6 males, 6 females) | Design: 2 groups (AD v Healthy) x 2 (lyrics sung v spoken).  Country: USA. Participants were tested individually in a single session lasting approximately 90 mins. Music lyrics were related to activities in daily life, e.g. the “Fill the pillbox” song). | Cognitive stimulation of learning new songs | Cognitive health (performance on neuropsychological tests). | This study with older adults with and without dementia sought to investigate the effects of music on memory for object words by making the lyrics of the songs relevant for the daily life of an older adult and by examining how musical encoding alters several different aspects of episodic memory. Participants performed better on a memory test of general lyric content for lyrics that were studied sung as compared with spoken. However, on a memory test of specific lyric content, participants performed equally well for sung vs spoken lyrics. |
| 59. Travis & Bowman, 2012 | N: 128 school and university students, Age: 13-23 years. Majority Latino/a (45.3%), African-American (26.6%) | Study design: cross-sectional survey to explore young people’s attitudes toward Hip-Hop culture, risky health behaviors, identity, criminal justice system. Country: USA. Setting: schools and university. | Music effects on ethnic identity and empowerment; self-esteem; social connections | Mental health (Depression) | Exposure to Hip Hop music was not significantly associated with undesirable outcomes (depression, low self-esteem). Positive ethnic identity was associated with greater music-influenced empowerment. Greater perceived music-influenced empowerment was associated with fewer depressive symptoms. Younger participants, who were male, who had high self-esteem, were most associated with perceived music-influenced risk. People who did not show depressive symptoms were more likely to feel that rap inspired them to connect with others, consider experiences of others, think critically about the world round them, and want to make a difference in their communities. |
| 60. Uhlig et al., 2019 | N: 160 year 8 students from a public school randomized; of whom 65 completed the Rap&Sing program and 30 completed control (classes as usual). Age: 9 - 13 years | Design: RCT, with Rap&Sing program or control; 2 time points (pre and post). Country: The Netherlands. Setting: public school. Evaluating the effects of Rap & Sing music program on measures of emotion regulation. Intervention ran weekly during class time for 4 months. | Emotion regulation; self-esteem; physical activity | Mental health protection; wellbeing | In the interviews, adolescents described the program as an “outlet”, and learned to be more “engaged in their emotions”, developed “self-knowledge”, “voiced their emotions into words”, and “were free to speak out”. Scores on the quantitative tests revealed increased problems and decreased self-esteem over time in the control group, which were not found in the Rap&Sing group. There was no effect of the program on students’ sleep - although a decline in total sleep time over the 4 months was smaller in the intervention group than in the control group. The results were interpreted as stabilizing adolescents’ emotion regulation and self-esteem, and protecting their wellbeing against potential deterioration during early adolescence. |
| ***Songwriting, Composition, Improvisation*** |  |  |  |  |  |
| 61. Bartleet et al., 2016 | N: unspecified university music students traveled to Central Australia to work alongside  Aboriginal and non-Indigenous musicians and artists on a range of community-led projects | Design: collective thematic analysis of data collected over 6 years of annual service learning trips. Country: Australia. Activities included jamming, songwriting, and performing. | Social and cultural connection and self-development | Cultural determinants of health (intercultural connection) | Through shared music making, the students experienced opportunities for social and intercultural connections or epiphanies that sparked lifelong journeys of intercultural development. This study culminated in a theoretical model with 3 ‘facings’ that occur during participation in the intercultural project: facing each other, facing others together, and facing ourselves. |
| 62. Fallon et al., 2020 | N: 105 university undergraduate students | Design: between groups. All participants completed a stressor task and were then assigned to one of three recovery conditions: control, music listening (ML), or music improvisation (MI) using a xylophone. Country: USA. | Physiological arousal measured by skin conductance (EDR); stress. | Satisfaction during recovery from a stressor. | Participants experienced a significant increase in stress response as a result of the stressor task. Although self-report measures indicated that mood improved during the recovery session, the MI group exhibited only differential effects for levels of satisfaction. The physiological data showed greater stress reduction for the ML condition compared to the MI and Control groups. |
| 63. Habron et al., 2013 | N: 6 older adults with previous music experience (3 males, 3 females). 3 living in the community and 3 in residential aged care. | Design: arts-based inquiry process; qualitative analysis. Country: England. Participants completed 6 full day sessions of composing music with a professional organization (Manchester Camerata) over a 2-month period | Meaningful occupation, control over musical materials, opportunities for creativity, social engagement with other participants and the musicians. | Wellbeing, healthy aging | The results emphasised occupation as essential to health and wellbeing in the later stages of life. The findings also highlight the use of music composition as a viable  arts-in-health occupation for older people and the arts-based research method of group composition. |
